# The oncogene *Etv5* promotes MET in somatic reprogramming and orchestrates epiblast/primitive endoderm specification during mESCs differentiation

**DOI:** 10.1038/s41419-018-0335-1

**Published:** 2018-02-14

**Authors:** Jinglong Zhang, Hongxia Cao, Jing Xie, Chen Fan, Youlong Xie, Xin He, Mingzhi Liao, Shiqiang Zhang, Huayan Wang

**Affiliations:** 1 College of Veterinary Medicine, Shaanxi Center of Stem Cells Engineering & Technology, Northwest A&F University, Yangling, Shaanxi 712100 China; 2 College of Life Sciences, Northwest A&F University, Yangling, Shaanxi 712100 China

## Abstract

Unipotent spermatogonial stem cells (SSCs) can be efficiently reprogrammed into pluripotent stem cells only by manipulating the culture condition, without introducing exogenous reprogramming factors. This phenotype raises the hypothesis that the endogenous transcription factors (TFs) in SSCs may facilitate reprogramming to acquire pluripotency. In this study, we screened a pool of SSCs TFs (*Bcl6b, Lhx1, Foxo1, Plzf, Id4, Taf4b*, and *Etv5*), and found that oncogene *Etv5* could dramatically increase the efficiency of induced pluripotent stem cells (iPSCs) generation when combined with Yamanaka factors. We also demonstrated that *Etv5* could promote mesenchymal-epithelial transition (MET) at the early stage of reprogramming by regulating *Tet2*-miR200s-*Zeb1* axis. In addition, *Etv5* knockdown in mouse embryonic stem cells (mESCs) could decrease the genomic 5hmC level by downregulating *Tet2*. Furthermore, the embryoid body assay revealed that *Etv5* could positively regulate primitive endoderm specification through regulating *Gata6* and negatively regulate epiblast specification by inhibiting *Fgf5* expression. In summary, our findings provide insights into understanding the regulation mechanisms of *Etv5* under the context of somatic reprogramming, mESCs maintenance, and differentiation.

## Introduction

Somatic cells can be reprogrammed into pluripotent state by overexpressing defined transcription factors (TFs) in vitro^1^. By contrast, spermatogonial stem cells (SSCs) endogenously express pluripotent TFs, including *Oct3/4*, *Sox2*, *Klf4*, *c-Myc,* and *Lin28*^2^. Therefore, SSCs can be efficiently reprogrammed into pluripotent state only by changing the culture condition without overexpressing exogenous TFs^3^. These evidences indicate the TFs involved in SSCs self-renewal may facilitate the reprogramming to acquire pluripotency. However, no study has been made systematically to investigate the roles of SSCs self-renewal TFs in somatic reprogramming.

Ets (E-twenty-six) variant gene 5 (*Etv5*) is one of the essential TFs for SSCs self-renewal^4,5^. *Etv5* knockout can cause Sertoli cell-only syndrome on mouse^4^. *Etv5* is also suggested to regulate proliferation and differentiation of mouse embryonic stem cells (mESCs)^6^. In addition, *Etv5* has been found as an early regulator during somatic reprogramming^7^. However, the exact mechanism of *Etv5* in mESCs and somatic reprogramming remains unclear.

Ten-eleven translocation family proteins (TET1/TET2/TET3) can oxidize 5-methylcytosine (5mC) into 5-hydroxymethylcytosine (5hmC) and regulate gene transcription^8^. Overexpression of *Tet1* and *Tet2* has proven to promote somatic reprogramming by reactivating pluripotency genes^9,10^. Ironically, *Tet1* and *Tet2* are dispensable for reprogramming SSCs into pluripotency^11^. However, *Tet1* and *Tet2* are suggested to erase H19 imprinting locus during reprogramming^11^. Although extensive studies have been taken to investigate the roles of Tets proteins, the upstream regulators of Tets proteins are not completely understood.

In this study, we screened a pool of SSCs TFs and found *Etv5* could remarkably promote the efficiency of reprogramming when combined with Yamanaka factors. We also proved that *Etv5* could facilitate mesenchymal–epithelial transition (MET) through *Tet2*-miR200s-*Zeb1* regulation axis. Moreover, we found *Etv5* knockdown (KD) could decrease the expression level of *Tet2* and 5hmC in mESCs and delayed the primitive endoderm differentiation by downregulating *Gata6*. In addition, *Etv5-KD* in mESCs could increase the epiblast specification in vitro by upregulating *Fgf5* expression. Our findings provide insights to understand the mechanisms of *Etv5* in somatic reprogramming, mESCs maintenance and differentiation.

## Results

### *Etv5* enhances somatic cell reprogramming

**Fig. 1:**
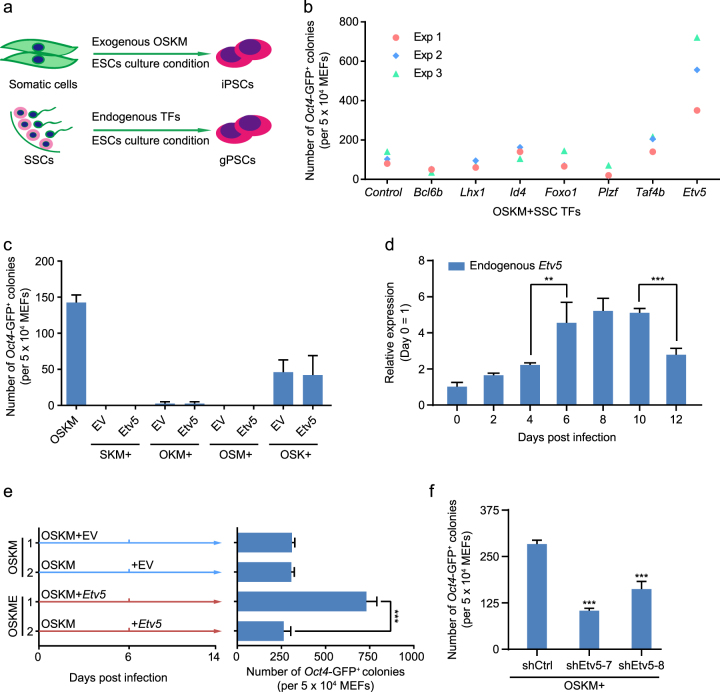
*Etv5* facilitates somatic cell reprogramming. **a** Diagram of iPSCs induction and germline-derived pluripotent stem cells (gPSC) induction. **b** Counts of *Oct4*-GFP^+^ colonies at 16 days post infection (dpi) from combinations of SSCs TFs with Yamanaka factor OSKM. The results of three independent experiments (Exp) were shown. The combination of Yamanaka factors OSKM with pMXs empty vector (EV) was used as control. **c** Counts of *Oct4*-GFP^+^ colonies at 16 dpi. OSKM was as positive control. The individual factor of OSMK was replaced by *Etv5*. The pMXs EV was used as vector control. **d** RT-qPCR analysis of endogenous *Etv5* in OSKM mediated cell reprogramming. **e** Counts of *Oct4*-GFP^+^ colonies at 14 dpi with two treatments. Introduction of *Etv5* at 0 and 6 dpi was compared. pMXs EV was used in parallel as vector control. **f** Counts of *Oct4*-GFP^+^ colonies at 16 dpi from combinations of OSKM with *Etv5* shRNA (shEtv5-7 and shEtv5-8). Nonsense shRNA (shCtrl) was used as vector control. Data are shown as mean ± SD, Two-way ANOVA with Dunnett’s multiple comparisons test was used for statistics analysis in **d** and **f**; Two-way ANOVA with Sidak’s multiple comparisons test was used for statistics analysis in **e**. **P < 0.01, ***P < 0.001 (*n* = 3)

We initially hypothesized that SSCs self-renewal TFs could promote the efficiency of generating induced pluripotent stem cells (iPSCs) (Fig. 1a). We screened SSCs-specific TFs by reference retrieval and constructed a pool consisting of *Bcl6b*, *Lhx1*, *Foxo1 Plzf*, *Id4*, *Taf4b,* and *Etv5*. Then we investigated their roles in promoting iPSCs efficiency. Combined with Yamanaka factors (*Oct4*, *Sox2*, *Klf4,* and *c-Myc*, abbreviated as OSKM hereafter), four SSCs TFs (*Bcl6b*, *Lhx1*, *Foxo1* and *Plzf*) decreased the efficiency of iPSCs (*Oct4*-GFP^+^ colonies/50000 MEFs). In contrast, three SSCs TFs (*Id4*, *Taf4b* and *Etv5*) increased the efficiency of iPSCs (Fig. 1b). Of note, Etv5 showed the highest efficiency when compared to *Id4* and *Taf4b*.

Then we tested whether the iPSCs generated from combination of OSKM and *Etv5* (abbreviated as OSKME-iPSCs hereafter) have typically pluripotent features. OSKME-iPSCs were positive for NANOG and SSEA-1 staining (Supplementary Fig. S1a) and expressed similar levels of endogenous pluripotent genes (*Oct4*, *Sox2*, *Nanog*, *Rex1*, and *Klf2*) when compared to OSKM-iPSCs and J1 mESCs (Supplementary Fig. S1b, c). All the characterized cell lines of OSKME-iPSCs were integrated exogenous *Oct4*, *Sox2*, *Klf4*, *c-Myc,* and *Etv5* (Supplementary Fig. S1d), but the transgenes were totally silenced (Supplementary Fig. S1e). The promoters of *Oct4* and *Nanog* also showed dramatic DNA demethylation in OSKME-iPSCs when compared with MEFs (Supplementary Fig. S1f). The OSKME-iPSCs could also differentiate into three germ layers as assayed by embryoid body (EB) differentiation (Supplementary Fig. S1g) and teratoma formation (Supplementary Fig. S1h).

We next examined whether *Etv5* could promote reprogramming when combined with OSK, but observed no significant improvement on iPSCs efficiency (Fig. 1c). Furthermore, we asked whether *Etv5* could replace anyone of other Yamanaka factors to achieve full reprogramming. However, *Etv5* was unable to replace either of them (Fig. 1c).

We also investigated whether endogenous *Etv5* was reactivated during OSKM-mediated reprogramming. Interestingly, a fluctuation of *Etv5* expression was observed in the process of reprogramming. Endogenous *Etv5* was activated on day 4 after introducing OSKM and reached the top level during day 6-10. Then the expression level of *Etv5* declined significantly (Fig. 1d). This phenotype intrigued us to determine the best time window of introducing exogenous *Etv5* to get the highest reprogramming efficiency. Therefore, we compared the reprogramming efficiency which was generated by two ways: introducing exogenous *Etv5* from beginning after OSKM transduction, and 6 days after OSKM transduction. Interestingly, we found overexpression of *Etv5* on day 6 after OSKM transduction could not increase the efficiency of reprogramming. Only overexpression of *Etv5* from beginning together with OSKM could significantly increase the iPSCs efficiency (Fig. 1e). To further determine whether endogenous *Etv5* is essentially required for reprogramming, we performed *Etv5* shRNA interference at the early stage of OSKM reprogramming, and found that *Oct4*-GFP^+^ colonies decreased significantly as the endogenous *Etv5* was knocked down (Fig. 1f). Together these results indicate that *Etv5* can increase iPSCs efficiency at the early stage of reprogramming when combined with Yamanaka factors.

### *Etv5* promotes cell reprogramming by facilitating MET

To dissect the mechanisms by which *Etv5* promotes reprogramming, we examined whether *Etv5* follows the proposed models at the early stage of reprogramming^12^. We considered two possibilities: (1) Whether *Etv5* could increase cell proliferation? (2) Whether *Etv5* could promote MET?

First, cell growth curves were drawn and compared between OSKME and OSKM. Unexpectedly, *Etv5* significantly decreased the cell proliferation (Supplementary Fig. S2a). This phenotype was consistent with the downregulation of cell cycle positive regulators (*Ccnd1*, *Ccne2,* and *Cdk4*) (Supplementary Fig. S2b) and the upregulation of cell cycle negative regulators (*Cdkn1a*, *Cdkn2a*) (Supplementary Fig. S2c) in OSKME when compared to OSKM.

**Fig. 2:**
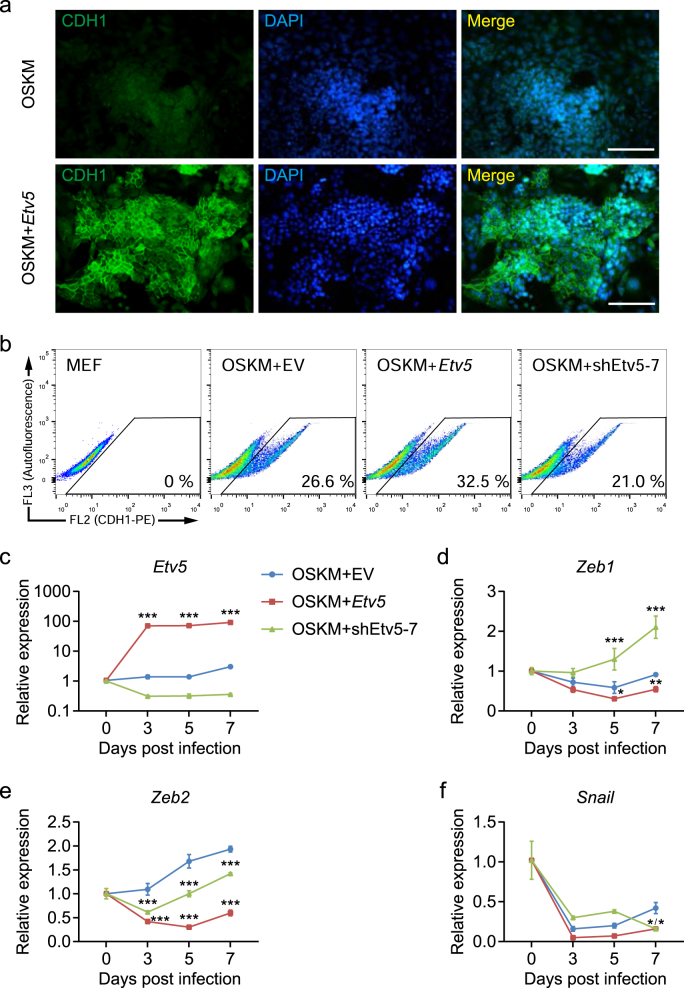
*Etv5* promotes somatic reprogramming by facilitating MET. **a** CDH1 immunofluorescence staining (green) of cells at 4 dpi with OSKME and OSKM. Nucleus was stained with DAPI (blue). Scale bars, 100 μm. **b** Flow cytometric analysis of CDH1 positive cells at 9 dpi. The cells infected with OSKM + EV, OSKM + *Etv5* and OSKM + shEtv5-7, respectively, were compared. MEFs were used as negative control. **c–f** RT-qPCR analysis of *Etv5*, *Zeb1*, *Zeb2,* and *Snail* at the early stage of reprogramming (Day 0–7). The cells infected with OSKM + EV, OSKM + *Etv5* and OSKM + shEtv5-7, respectively, were compared. Two-way ANOVA with Dunnett’s multiple comparisons test was used. Data are shown as mean ± SD (*n* = 3). OSKM + EV was set as the control. *P < 0.05, **P < 0.01, ***P < 0.001

Second, we assessed if *Etv5* could promote MET during the early stage of reprogramming. Immunofluorescence staining of infected cells (4 days post infection, 4 dpi) revealed that CDH1 positive cells were found more in OSKME when compared that with OSKM (Fig. 2a). Flow cytometry analysis of infected cells (9 dpi) also confirmed that OSKME significantly increased CDH1 positive cells when compared that with OSKM. By contrast, endogenous *Etv5* KD in OSKM combination decreased CDH1 positive cells (Fig. 2b). Furthermore, we investigated the time course expression of *Etv5* and mesenchymal TFs (*Zeb1*, *Zeb2,* and *Snail*) at the early stage of reprogramming (Fig. 2c-f). We found that OSKME significantly decreased the expression levels of *Snail*, *Zeb1,* and *Zeb2* when compared that with OSKM. However, *Etv5* KD in OSKM only exclusively increased the expression level of *Zeb1* (Fig. 2d). Together these results indicate that *Etv5* could promote MET by decreasing *Zeb1* expression.

### *Etv5* promotes MET through *Tet2*-miR200s-*Zeb1* axis

**Fig. 3:**
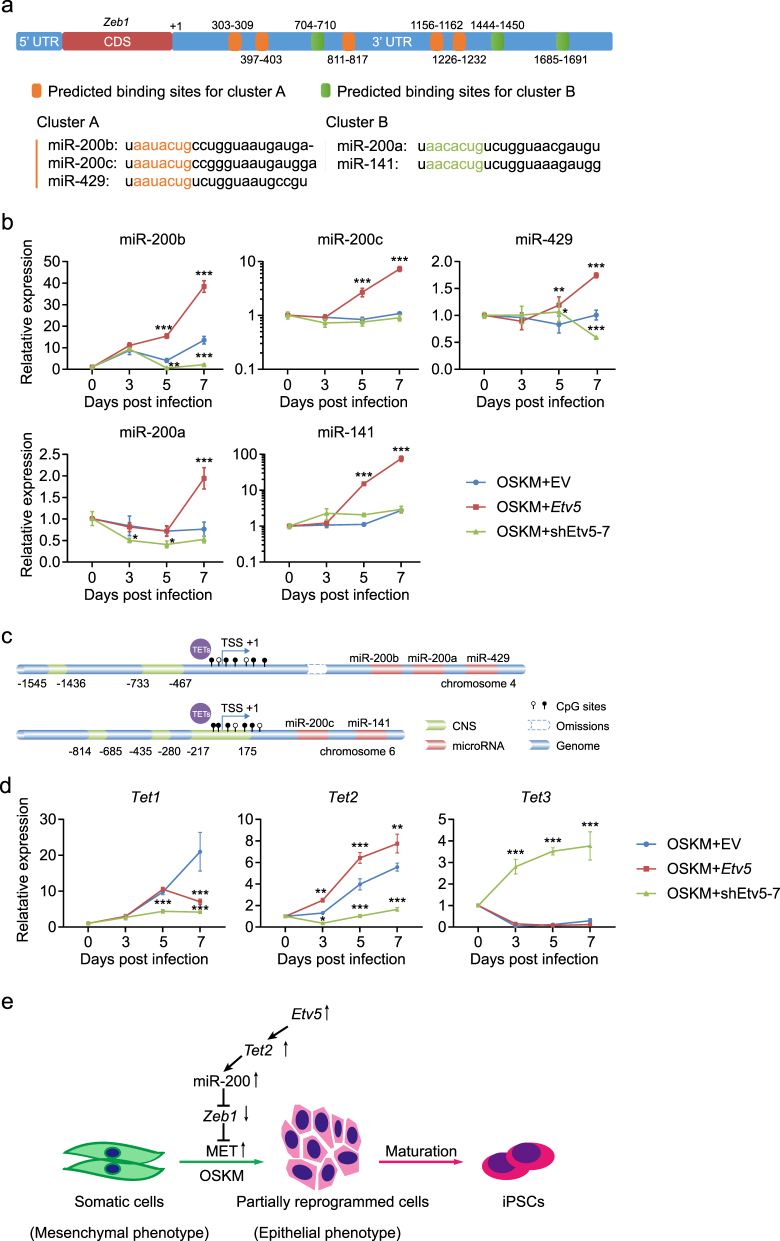
*Etv5* promotes MET through *Tet2*-miR-200s-*Zeb1* axis. **a** Predicted binding sites of miR-200 family located in 3′ UTR of *Zeb1*. Five sites (orange) are supposed to be targeted by cluster A of miR-200 family (miR-200b,-200c and -429). Three sites (green) are supposed to be targeted by cluster B of miR-200 family (miR-200a, and -141). **b** RT-qPCR analysis of miR-200 family members during the early stage of reprogramming (Day 0–7). Cells infected with OSKM + EV, OSKM + *Etv5* and OSKM + shEtv5-7, respectively, were compared. Two-way ANOVA with Dunnett’s multiple comparisons test was used. Data are shown as mean ± SD (*n* = 3), OSKM + EV was set as the control, *P < 0.05, **P < 0.01, ***P < 0.001. **c** The diagram of genomic structure of miR-200 family on chromosome 4 and chromosome 6. The red regions represent miRNA clusters and green regions represent conserved noncoding sequence (CNS) across mammals. The differentially methylated region around transcription start site (TSS) of miR-200s family is suggested to be demethylated by TET proteins (TET1, TET2, and TET3). **d** RT-qPCR analysis of *Tet1*, *Tet2.* and *Tet3* during the early stage of reprogramming (Day 0–7). The cells infected with OSKM + EV, OSKM + *Etv5* and OSKM + shEtv5-7, respectively, were compared. Two-way ANOVA with Dunnett’s multiple comparisons test was used. Data are shown as mean ± SD (*n* = 3), OSKM + EV was set as the control, *P < 0.05, **P < 0.01, ***P < 0.001. **e** The working model of *Etv5* in somatic reprogramming. Overexpression (↑) of *Etv5* with Yamanaka factor (OSKM) can facilitate MET through *Tet2*-miR-200s-*Zeb1* axis at the early stage of cell reprogramming

Interactions between Zeb family (Zeb1 and Zeb2) and miR-200s family are suggested to regulate MET and epithelial-mesenchymal transition (EMT) in development, tumorigenesis, and reprogramming^13,14^. So we asked whether Zeb1 interacts with miR-200s family members during OSKME-mediated reprogramming. We first analyzed the complementation of 3′ UTR of *Zeb1* mRNA and miRNA-200 family members. Interestingly, there are five sites in 3′ UTR of *Zeb1* potentially targeted by cluster A of miRNA-200 family members (miR-200b,-200c, and -429) and three sites by cluster B of miRNA-200 family members (miR-200a and -141) (Fig. 3a). We then assessed whether overexpression of *Etv5* could increase expression levels of any miRNA-200 family members. Expectedly, all the miRNA-200 family members were found to be expressed with higher expression levels in OSKME combination when compared that with OSKM during the first seven days of reprogramming (Fig. 3b). However, *Etv5* KD in OSKM combination only led to the decreased expression levels of miR-200a, miR-200b, and miR-429 (Fig. 3b).

Next, we asked whether *Etv5* could directly regulate miR-200s family members during OSKME-mediated reprogramming. We analyzed the promoter regions (3.5 kb fragment upstream of transcription start site (TSS)) of miR-200s family members using online genomics comparative tool VISTA. Conserved noncoding sequences (CNS) were found across mammal species (Fig. 3c). However, we found no potential ETV5 binding sites located in these CNS regions. Alternatively, Tet proteins (TET1, TET2, and TET3) were proposed to directly regulate the differentially methylated region around TSS of miR-200s family^15^ (Fig. 3c). Therefore, we asked whether overexpression of *Etv5* could increase the expression levels of Tet family members. Interestingly, only *Tet2* was found to be expressed with higher expression levels in OSKME combination when compared that with OSKM. *Etv5* KD in OSKM also confirmed the exclusive contribution of *Etv5* to *Tet2* expression (Fig. 3d). Next, we performed luciferase assay to determine whether *Etv5* could directly activate *Tet2* promoter or enhancer^16^. Two genomic fragments (Pro I and Pro II) with predicted *Etv5* binding site showed promoter activity in NIH 3T3 cells, although the activities varied a lot (Supplementary Fig. S3a, b). Then we co-transfected these constructs with *Etv5* overexpression vectors, but found no significant increase of luciferase expression for both Pro I and Pro II when compared with control (Supplementary Fig. S3c). Similar result was observed for fragment En I when comparing its enhancer activities between *Etv5* and GFP overexpression conditions (Supplementary Fig. S3c). Together these results suggest that *Etv5* may regulate *Tet2* indirectly or involve of additional co-factors.

Collectively, the results above suggest that *Etv5* can promote MET through *Tet2*-miR200s-*Zeb1* axis (Fig. 3e).

### *Etv5* KD decreases 5hmC level by downregulating *Tet2* in mESCs

**Fig. 4:**
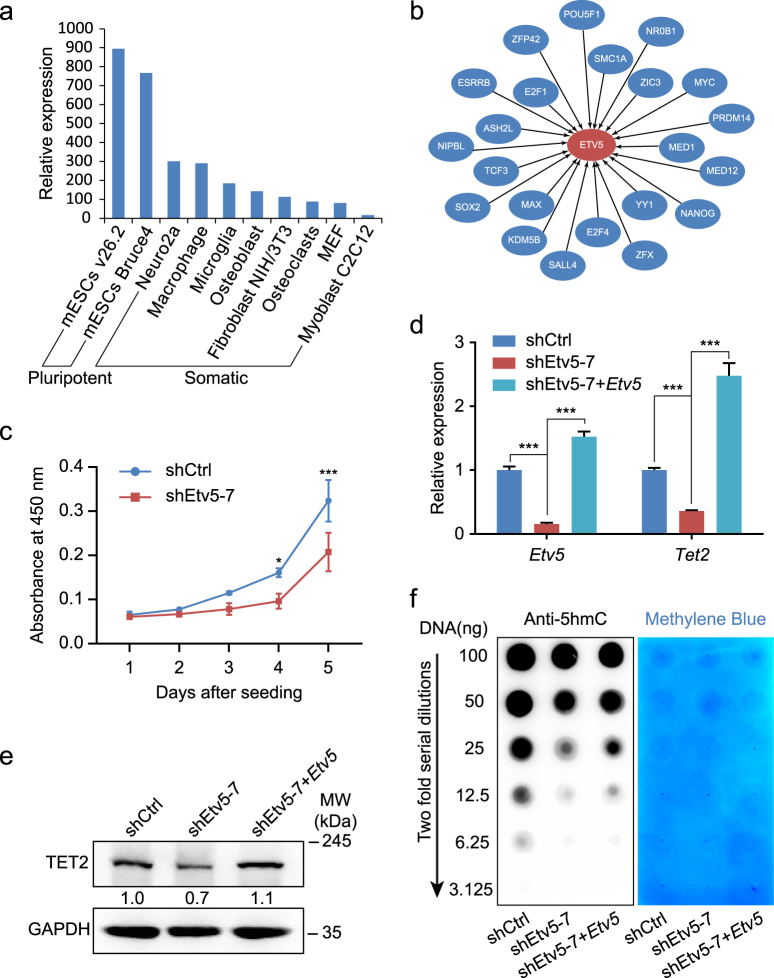
*Etv5* positively regulates *Tet2* but is dispensable for mESCs maintenance. **a** Comparison of *Etv5* expression levels between mESC lines and somatic cell lines. The relative expression was based on the microarray data from BioGPS database. **b** The interactions between pluripotency relevant regulators and *Etv5*. ChIP-seq and ChIP-chip data with *Etv5* as target were extracted from ESCAPE database and used for drawing these interactions. **c** Growth curve of J1 mESCs stably infected with shCtrl and *Etv5* shRNA (shEtv5-7). **d** RT-qPCR analysis of *Etv5* and *Tet2* in mESCs stably infected with shCtrl, *Etv5* shRNA (shEtv5-7), and shEtv5-7 plus lentiviral *Etv5*. Data are shown as mean ± SD (*n* = 3). *P < 0.05, ***P < 0.001. Two-way ANOVA with Sidak’s multiple comparisons test was used for **c** . One-way ANOVA with Dunnett’s multiple comparisons test for **d** . **e** Western blotting of TET2 in mESCs stably infected with shCtrl, shEtv5-7, and shEtv5-7 + *Etv5*. GAPDH was used as internal control. The relative quantification is also shown. **f** Dot blot of global 5hmC in mESCs stably infected with shCtrl, shEtv5-7, and shEtv5-7 plus lentiviral *Etv5*. The blotting result of serially diluted genomic DNA (100-3.125 ng) was shown (left panel). The same membrane stained with methylene blue as DNA loading control was also presented (right panel)

The beneficial effect of *Etv5* in somatic reprogramming intrigued us to investigate the roles of *Etv5* in mESCs maintenance. We firstly analyzed the expression levels of *Etv5* among different cell types from BioGPS database, and found mESCs expressed the highest level of *Etv5* when compared to somatic cells (Fig. 4a). We also analyzed the ChIP-seq and ChIP-chip data from ESCAPE database and constructed the interaction network between well-known pluripotent factors and *Etv5* (Fig. 4b). *Etv5* is potentially regulated by 22 pluripotency relevant regulators, including POU5F1, SOX2, NANOG, ESRRB, SALL4, PRDM14, TCF3, MYC, NR0B1, YY1, ZFX, SMC1A, KDM5B, E2F1, E2F4, ZFP42, MAX, NIPBL, ASH2L, MED1, MED12, and ZIC3 (Fig. 4b). These analyses suggest *Etv5* to be an important member of pluripotency regulation network.

Then we infected J1 mESCs with *Etv5* shRNA with puromycin resistance and tried to examine the phenotype of *Etv5*-KD mESCs. The efficiency of *Etv5* knockdown was confirmed by RT-qPCR (Supplementary Fig. S4a). Compared to shEtv5-8, the shEtv5-7 showed the highest efficiency to downregulate *Etv5*. So we focused on shEtv5-7 and used it in the following assays. Although *Etv5-KD* colonies became looser in KSR + LIF medium, they could recover their compact morphology in 2i medium (Supplementary Fig. S4b).

To evaluate the effect of *Etv5* KD on self-renewal of mESCs, we compared the proliferation and pluripotency genes expression between *Etv5-KD* and nonsense shRNA control colonies (abbreviated as shCtrl colonies hereafter). Compared to shCtrl colonies, the proliferation rate of *Etv5-KD* colonies was lowered (Fig. 4c). However, there were no obvious differences between shCtrl colonies and *Etv5-KD* colonies on alkaline phosphatase staining and immunofluorescence of OCT4, SOX2, and SSEA-1 (Supplementary Fig. S4c). These results indicate that *Etv5* is dispensable for pluripotency maintenance although *Etv5-KD* colonies compromise their proliferation.

We next asked whether *Etv5* could positively regulate *Tet2* in mESCs as seen in somatic reprogramming. RT-qPCR and Western blotting both showed that *Tet2* was significantly downregulated in *Etv5-KD* colonies (Fig. 4d,e). Furthermore, we did dot blot experiment for 5hmC and found the genome 5hmC level was expectedly decreased as *Etv5* was downregulated (Fig. 4f). The downregulation of *Tet2* and genomic 5hmC level in *Etv5*-KD mESCs was rescued by re-introducing *Etv5* (Fig. 4d-f). Collectively, these results indicate that *Etv5* can positively regulate *Tet2* expression and thus influence the genomic 5hmC level in mESCs.

### Transcriptome changes caused by *Etv5* KD in mESCs

**Fig. 5:**
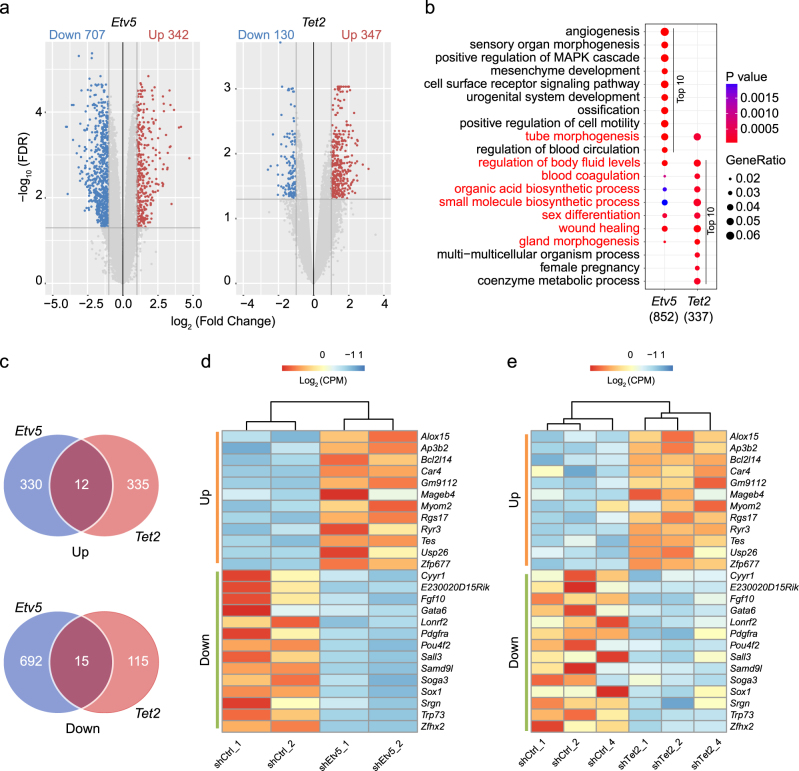
Integration analysis of RNA-seq data from *Etv5*-KD and *Tet2*-KD mESCs. **a** Volcano plot of differentially expressed genes generated from *Etv5*-KD RNA-seq data and *Tet2*-KD RNA-seq data. The differentially expressed genes with FDR < 0.05 and log_2_ (fold change) >1 are shown. The numbers of downregulated genes (blue) and upregulated genes (red) are shown on the top. **b** GO analysis of differentially expressed genes in *Etv5*-KD mESCs (852 annotated genes, left column) and *Tet2*-KD mESCs (337 annotated genes, right column). The top 10 GO terms of biological processes were plotted for *Etv5*-KD mESCs and *Tet2*-KD mESCs from each end of the graph according to their *P* values ranking. The GO terms from the other group overlapped with the top 10 GO terms were also plotted and marked in red. The color of circles indicates the *P* value. The size of circles indicates the gene ratio. **c** Venn analysis of differentially expressed genes from *Etv5*-KD mESCs, and *Tet2*-KD mESCs. The upregulated and downregulated genes are compared and shown, respectively. **d**, **e** Heatmaps showing detailed expression information of the commonly upregulated and downregulated genes in *Etv5*-KD mESCs (**d**) and *Tet2*-KD mESCs (**e**). CPM, counts per million. The color scale represents the value of log_2_ (CPM).

The paradoxical findings of *Etv5* in mESCs maintenance prompted us to hypothesize that *Etv5* may function mainly in mESCs differentiation. We then profiled the genes upregulated and downregulated in *Etv5-KD* mESCs by RNA-seq. Of note, 1049 genes were found to be significantly influenced by *Etv5* KD (log_2_ FC > 1, FDR < 0.05) (Fig. 5a, left). Gene ontology (GO) analysis revealed that these differentially expressed genes showed high enrichment in GO terms like angiogenesis, MAPK signaling pathway, urogenital system development and sensory organ (eye) morphogenesis (Fig. 5b). To further explore the potential biological effects caused by *Etv5* through *Tet2*, We carried out paralleled analysis of RNA-seq data from *Tet2*-KD mESCs and their corresponding shCtrl mESCs^17^. There were five original pairs (*Tet2*-KD versus shCtrl) of RNA-Seq replicates and principal component analysis (PCA) revealed poor consistency of pair 3 and pair 5 (Supplementary Fig. S5a). Therefore, we used the filtered pairs (1, 2, and 4) for the subsequent analysis (Supplementary Fig. S5b). Interestingly, only 477 genes were found to be differentially expressed between *Tet2*-KD and shCtrl mESCs (log_2_ FC > 1, FDR < 0.05) (Fig. 5a, right) and these genes were highly enriched in biological process like gland morphogenesis (predominantly liver and prostate), wound healing, blood coagulation, and small molecule biosynthetic process (Fig. 5b and Supplementary Fig. S5c-e). Of note, there is broad overlap of *Tet2* GO terms with that of *Etv5*, suggesting that multiple biological effects of *Etv5* may be mediated through *Tet2*.

Furthermore, we asked what genes were commonly regulated by both *Etv5* and *Tet2*. Venn analysis revealed that 12 genes were commonly upregulated and 15 genes were commonly downregulated in both *Etv5*-KD and *Tet2*-KD mESCs (Fig. 5c). Heatmap analysis of these commonly changed genes in *Etv5*-KD and *Tet2*-KD mESCs were shown in Fig. 5d and Fig. 5e, respectively.

Briefly, the transcriptome analysis indicates that *Etv5* and *Tet2* have overlapped functions in regulating mESCs differentiation. Since *Etv5* could function upstream to positively regulate *Tet2* as demonstrated before, these common differentially expressed genes are supposed to be downstream targets of *Etv5-Tet2* regulation axis.

### *Etv5* orchestrates the specification of primitive endoderm and epiblast during mESCs differentiation in vitro

**Fig. 6:**
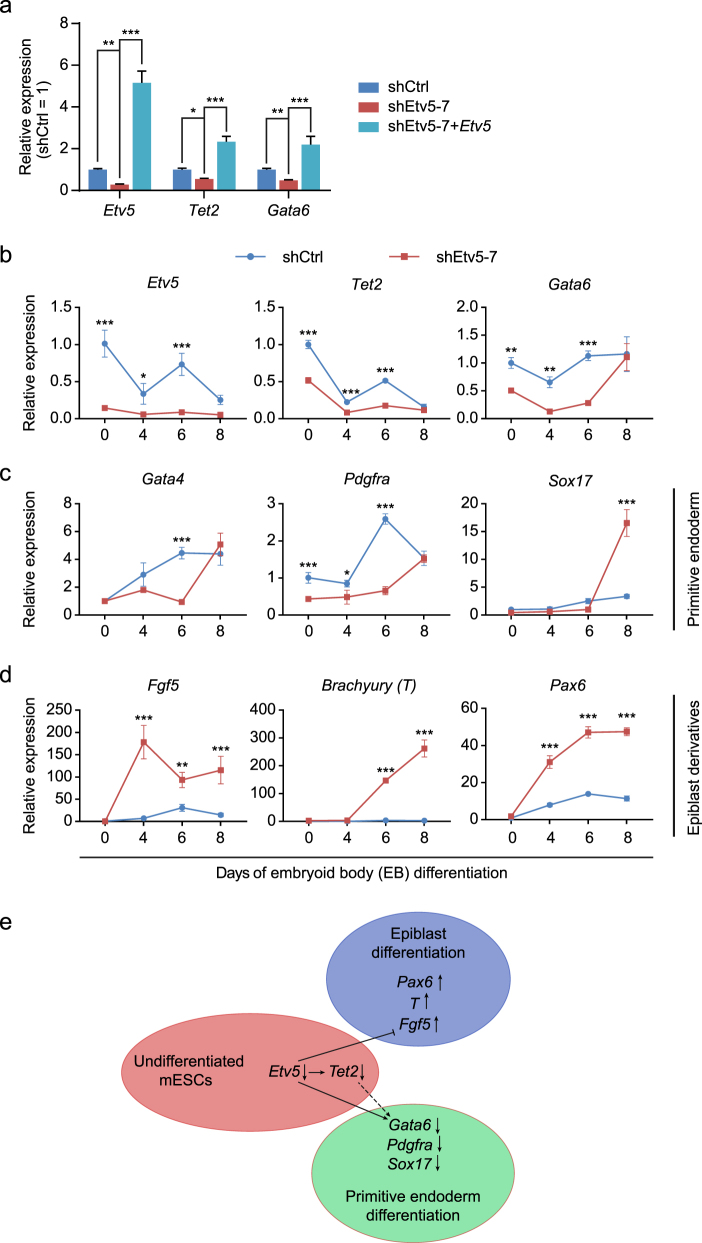
*Etv5* orchestrates the specification of primitive endoderm and epiblast during mESCs differentiation in vitro. **a** RT-qPCR verification of the positive regulation relationship among *Etv5*, *Tet2,* and *Gata6* in undifferentiated mESCs. The mESCs stably infected with nonsense shCtrl, shEtv5-7, and shEtv5-7 plus lentiviral *Etv5* are compared. One-way ANOVA with Dunnett’s multiple comparisons test was used. Data are shown as mean ± SD (*n* = 3). *P < 0.05, **P < 0.01, ***P < 0.001. **b** RT-qPCR analysis of *Etv5*, *Tet2,* and *Gata6* during mESCs differentiation in vitro. **c** RT-qPCR analysis of of primitive endoderm marker genes (*Gata4*, *Pdgfra,* and *Sox17*) during mESCs differentiation in vitro. **d** RT-qPCR analysis of epiblast marker gene and its derivatives marker genes during mESCs differentiation in vitro. The expression of *Fgf5* (epiblast), *T* (mesoendoderm) and *Pax6* (ectoderm) was observed. The mESCs stably infected with shCtrl and shEtv5-7 were used for EB differentiation and compared in **b**–**d**. Data are shown as mean ± SD (*n* = 3). Two-way ANOVA with Sidak’s multiple comparisons test was used in **b**–**d**. *P < 0.05, **P < 0.01, ***P < 0.001. **e** The working model of *Etv5* in mESCs differentiation. *Etv5* knockdown (↓) can delay the primitive endoderm specification by downregulating *Gata6*. In parallel, *Etv5* knockdown can promote specification of epiblast and its derivative mesoendoderm and ectoderm

Among the 15 commonly downregulated genes, there were two genes (*Gata6* and *Pdgfra*) which were proposed to regulate primitive endoderm differentiation in the second fate determination of late blastocyst^18^. Therefore, we speculated that *Etv5*-*Tet2* regulation axis may facilitate primitive endoderm differentiation by maintaining *Gata6* or *Pdgfra* at a poised state in mESCs. *Gata6* is a master TF of primitive endoderm differentiation^18^, so we focused on it and did RT-qPCR validation. As *Etv5* was knocked down in mESCs, both *Tet2* and *Gata6* were similarly downregulated (Fig. 6a), which was consistent to the results as observed in RNA-seq (Fig. 5d,e). Even more, we demonstrated that the downregulation of *Tet2* and *Gata6* in *Etv5*-KD mESCs was recovered as *Etv5* was re-introduced (Fig. 6a). Together these findings indicate that *Etv5* may positively function upstream of *Tet2* which further positively regulates *Gata6* at a poised state in undifferentiated mESCs.

To examine whether the relationship of positive regulation among *Etv5*, *Tet2,* and *Gata6* also exists as mESCs differentiated, we investigated the time course expression of them during EB differentiation (Fig. 6b). Interestingly, we found that there was a fluctuation of *Etv5* expression during an eight-day EB differentiation (Fig. 6b, left). According to the fluctuation of *Etv5* expression level, we divided the eight days of EB differentiation into three phases: phase 1 (Day 0–4), the relative expression level (REL) of *Etv5* dropped from 1 to ~0.3; phase 2 (Day 4–6), the REL of *Etv5* upregulated from ~0.3 to ~0.7; phase 3 (Day 6–8), the REL of *Etv5* then dropped from ~0.7 to ~0.2. Surprisingly, the fluctuation trend of *Tet2* and *Gata6* was highly matched to the fluctuation expression of *Etv5* from phase 1 to 3 (Fig. 6b, middle and right). The REL of *Etv5* in *Etv5*-KD mESCs showed similar fluctuation pattern, but at quite low level during the whole process of EB differentiation in parallel (Fig. 6b, left). Expectedly, the REL of *Tet2* and *Gata6* in *Etv5*-KD EB was significantly lower than that in shCtrl EB at the end of phase 1 and phase 2 (Fig. 6b, middle and right). Due to the similarly low level of *Etv5* between shCtrl EB and *Etv5*-KD EB at the end of phase 3, there was no significant difference on the REL of *Tet2* and *Gata6* at this time point (Fig. 6b). Collectively, these results suggest that the relationship of positive regulation among *Etv5*, *Tet2,* and *Gata6* faithfully exists during mESCs differentiation.

These findings above intrigued us to investigate whether *Etv5* KD can lead to a delayed primitive endoderm differentiation. Although no obvious morphology difference was found between shCtrl EB and *Etv5*-KD EB (Fig. S6), the REL of primitive endoderm marker genes (*Gata4* and *Pdgfra*) in *Etv5-*KD EB was significantly lower than that in shCtrl EB at the end of phase 1 and phase 2 (Fig. 6c, left and middle). Unexpectedly, primitive endoderm marker gene *Sox17* showed slight expression difference between shCtrl EB and *Etv5*-KD EB at the end of phase 1 and phase 2 (Fig. 6c, right). Interestingly, *Sox17* expression in *Etv5*-KD EB increased dramatically at the end of phase 3 and exceeded its expression in shCtrl EB (Fig. 6c, right). Summarily, these EB results indicate that primitive endoderm specification is supposed to be positively regulated by *Etv5*-*Tet2*-*Gata6* axis during mESCs differentiation in vitro.

Binary segregation of inner cell mass into primitive endoderm and epiblast is regarded as the second fate decision of preimplantation embryo^19^. So we further asked whether *Etv5* can negatively regulate the epiblast differentiation. We observed the expression trend of *Fgf5*, one master gene of epiblast, during EB differentiation. Surprisingly, the REL of *Fgf5* in *Etv5*-KD EB was significantly higher than that in shCtrl EB from phase 1 to 3 (Fig. 6d, left). As the preimplantation embryo further progresses, the epiblast is believed to specialize into mesoendoderm and ectoderm^20^. So we tested whether the expression of mesoendoderm marker gene *Brachyury* (*T*) and ectoderm marker gene (*Pax6*) increased as *Fgf5* did. Expectedly, the REL of *T* and *Pax6* in *Etv5*-KD EB was both significantly higher than that in shCtrl EB during the whole process of differentiation (Fig. 6d, middle and right). Briefly, the specification of epiblast and its derivatives is supposed to be negatively regulated by *Etv5* during mESCs differentiation in vitro.

Taken together, these EB differentiation results indicate that *Etv5* mainly functions to orchestrate the specification of primitive endoderm and epiblast during mESCs differentiation in vitro (Fig. 6e).

## Discussion

In this study, we demonstrated that *Etv5* could promote MET at the early stage of reprogramming through *Tet2*-miR200-*Zeb1* regulation axis, which further increased the final efficiency of iPSCs induction (Fig. 3e). In addition, Etv5 KD in mESCs could decrease the genomic 5hmC level by downregulating Tet2. Moreover, *Etv5* could orchestrate the specification of primitive endoderm and epiblast during mESCs differentiation in vitro (Fig. 6e). Our findings indicate the novel mechanisms of *Etv5* in somatic reprogramming, mESCs maintenance, and differentiation.

*Etv5* was found to be upregulated in the early stage of OSKM-mediated reprogramming^7^. Our study confirmed this finding and further revealed that *Etv5* could promote reprogramming efficiency when combined with OSKM. By contrast, *Etv5* could not increase reprogramming efficiency when combined with OSK (Fig. 1c). These findings suggest that cooperation between *Etv5* and *c-Myc* may be essentially required for *Etv5* to promote reprogramming. Interconnectivity analysis of pluripotent TFs in human ESCs indicates ETV5 as a co-regulator of c-MYC^21^. Further study of interactions between *Etv5* and *c-Myc* may provide insights into the mechanism of OSKME-mediated reprogramming.

The process of reprogramming MEFs into iPSCs is generally divided into early, middle, and late stage^12^. One of the critical features in early stage of reprograming is MET, which is believed to be orchestrated by TFs, epigenetic modifiers, and signaling pathways^22,23^. This is exemplified by the finding that Tet proteins and TDG glycosylase can mediate DNA demethylation and reactivation of miRNAs critical for MET in OSKM-mediated reprogramming^15^. In OSKME-mediated reprogramming, *Etv5* could contribute exclusively the improvement of MET by *Tet2*-miR200s-*Zeb1* regulation axis. This reflects a cooperation of transcriptional regulation and posttranscriptional regulation during the process of MET. We also noted that *Etv5* KD in OSKM could not completely block MET, which indicated additional regulation axis must contribute the MET at the early stage of reprogramming. This speculation is consistent with the observation that *Oct4*/*Sox2* can induce MET by miR-200-*Zeb2* pathway^14^. In addition, BMPs molecules in the serum-containing mESCs medium may also facilitate MET in OSKME-mediated reprogramming^24^.

*Etv5* was once predicted as an important member of mESCs pluripotency network^25^. ChIP-seq and ChIP-chip analysis of classical pluripotency relevant TFs also support the view that *Etv5* is integrated into the regulatory network of pluripotency (Fig. 4b). Ironically, we observed no dramatic changes on colony morphology and expression level of classical pluripotency TFs in *Etv5-KD* mESCs. This phenotype may be partially explained by the functional redundancy between *Etv4* and *Etv5* in mESCs^6^.

By contrast, *Etv5* orchestrated the specification of primitive endoderm and epiblast during mESCs differentiation in vitro. These findings provide complementary evidences to understand the roles of *Etv5* in fine tuning cell fate determination of primitive endoderm and epiblast in vivo^26^. Interestingly, *Etv5* is a direct target of *Gata6* during primitive endoderm differentiation^27^. Therefore, it is likely that the primitive endoderm specification is controlled by a negative feedback between *Etv5* and *Gata6*. Whether there is a direct interaction of *Tet2* and *Gata6* remains to be examined in the future.

Collectively, our findings in this study provide insights into understanding the mechanisms of *Etv5* in somatic reprogramming, mESCs maintenance and differentiation in vitro.

## Materials and methods

### Cell culture

Mouse embryonic fibroblasts (MEFs) were derived from 13.5 d.p.c mouse embryos by crossing male ICR mice to female OG2 mice (B6;CBA-Tg(Pou5f1-EGFP) 2Mnn/J). Feeder cells were prepared by treating MEFs with 10 μg ml^−1^ mitomycin C (Sigma). MEFs were maintained in high glucose DMEM (Hyclone) supplemented with 15% FBS (Gibco) and 0.1 mM non-essential amino acids (Gibco). HEK293T and Plat-E cells were maintained in DMEM supplemented with 10% FBS. For routine culture, iPSCs and J1 (129/Sv) mESCs were maintained on feeder layers in standard mESCs medium. The standard mESCs medium was composed of high glucose DMEM, 15% FBS, 100 μM non-essential amino acids, 1 mM L-glutamine (Gibco), 100 μM β-mercaptoethanol (Gibco), 100 μg ml^−^^1^ Vitamin C (Sigma) and 1000 units ml^−^^1^ leukaemia inhibitory factor (LIF) (Millipore). For gene knockdown and differentiation experiments, J1 mESCs were maintained in feeder-free conditions on 0.1% gelatin-coated tissue culture plates with KSR based mESCs medium which was modified from standard mESCs medium. The component of 15% FBS in standard mESCs medium was replaced by 12% knockout replacement serum (KSR) (Gibco) plus 3% FBS. To compare the pluripotent gene expression of iPSCs under different culture conditions, pluripotent cells maintained in standard mESCs medium and 2i medium were used. The 2i medium consists of N2B27 medium, 100 μM β-mercaptoethanol, 1 μM PD0325901 (StemRD), 3 μM CHIR99021 (StemRD) and 1000 U ml^−^^1^LIF. The medium described above was changed daily and all the cells lines were routinely tested to keep mycoplasma free.

### Plasmid construction

For the package of retrovirus carrying SSCs specific TFs, coding sequences (CDS) of candidate TFs were amplified from cDNA library of mouse testis and cloned into the pMXs vector. The lentiviral vector pTrip-CAGG-*Etv5*-IRES-Neo was constructed by replacing puro cassette in pTrip-CAGG-MCS-IRES-puro plasmid with NeoR gene first and followed by insertion of *Etv5* CDS. pSicoR-shEtv5-7-puro, pSicoR-shEtv5-8-puro, and control vector pSicoR-shCtrl-puro are kind gifts from Dr. Marius Wernig and designed to target the 3′ UTR of *Etv5*. The specificity of these *Etv5* shRNAs vectors had been validated in the publication by Lujan E et al.^7^. For reporter plasmid construction, putative promoter fragments were amplified from MEFs genomic DNA using Phanta Max Super-Fidelity DNA Polymerase (Vazyme) and subcloned into the multiple cloning sites of pGL3-Basic vector (Promega). Putative enhancer fragment were subcloned into the pGL3-Promoter vector (Promega) containing a minimal simian virus40 (SV40) promoter at the MluI/XhoI sites. All the plasmids described above were verified by sequencing and primers involved are listed in Table S1.

### Dual-luciferase reporter assay

One day before the transfection, NIH 3T3 cells were seeded at 2.5 × 10^4^ cells per well of a 24-well plate. All pGL3 constructs were co-transfected with pRL-TK and pMXs-*Etv5* using lipofectamine 2000 (Invitrogen). Cells were harvested 2 days after transfection for measurement of luciferase activities by using Dual-luciferase reporter assay system (Promega). Three independent transfections in triplicate were performed for the luciferase assay.

### Mouse iPSCs induction

Mouse iPSCs were generated with retroviruses as described previously^1^ with some modifications. Briefly, Plat-E cells were seeded at a density of 6 × 10^6^ cells per 100-mm culture dish one day before the transfection. The pMXs-based retroviral vectors (20 μg) were transfected into Plat-E cells using Calcium Phosphate Cell Transfection Kit (Beyotime Biotechnology) according to the manufacturer’s instruction. The medium was changed 12 h later. Virus-containing supernatants were collected twice at 48 h and 72 h post transfection, and filtered through 0.45 μM PVDF filter (Millipore). Polybrene (Sigma) was added to the supernatant at a final concentration of 8 μg ml^−^^1^ before the cocktail of TFs retroviruses was added to the MEFs. The MEFs within three passages were seeded at 3.5 × 10^4^ cells per well in a 6-well plate the day before transduction, and incubated with the retrovirus supernatant for 12 h. After two rounds of infection, the medium was changed to standard mESCs medium and changed daily. *Oct4*-GFP^+^ colonies were counted on indicated days, after which colonies were picked up for expansion. For induction of iPSCs from *Etv5*-shRNA infected MEFs, puromycin (2 μg ml^−^^1^) was added to the standard mESCs medium after final round of infection and removed 3 days later.

### Immunofluorescence staining

The mESCs colonies were fixed with 4% PFA for 20 min at room temperature, washed three times with PBS and permeabilized with 0.1% Triton X-100 at room temperature for 20 min. After three washes with PBS, the cells were blocked with 5% FBS for one hour at room temperature and incubated with diluted primary antibody overnight. The next day, the cells were washed three times with PBS and incubated with fluorophore-conjugated secondary antibody for one hour at room temperature and followed by DAPI staining. The images were taken under the fluorescence microscope (EVOS FL, Thermo Scientific). The following antibodies were employed in this study: anti-SSEA1 (1:100, 4744s, CST), anti-Nanog (1:300, ab80892, Abcam), anti-Oct-3/4 (1:200, sc-5279, Santa Cruz), anti-E-cadherin/CDH1 (1:1000, sc-8426, Santa Cruz), anti-Neuronal Class III β-Tubulin (1:1000, MMS-435P, Covance), anti-DESMIN (1:500, MAB3430, Millipore), anti-AFP (1:50, MAB1368, R&D System).

### Transgenes integration and exogenous transgenes silencing

For transgenes integration detection, genomic DNA of mouse iPSCs was extracted using TIANamp Genomic DNA Kit (TIANGEN). For exogenous transgenes silencing detection, cDNA library was prepared as described below. PCR reactions were conducted using Taq DNA Polymerase (Fermentas) on S1000 Thermal Cyclers (Bio-Rad). Primers employed in this section were listed in Table S1.

### Reverse transcription PCR and real-time PCR

Total RNA was extracted using RNAiso Plus (TaKaRa) according to the manufacturer’s instruction. First strand cDNA synthesis was conducted using RevertAid First Strand cDNA Synthesis Kit (Thermo Scientific), during which the genomic DNA was removed with DNase I (Thermo Scientific) treatment. For the quantification of mRNA expression, a mixture of oligo (dT)18 primer and random hexamer primer was employed in the reverse transcription reaction and quantitative real-time PCR was performed using TransStart® Tip Green qPCR SuperMix (TansGen Biotech) on StepOnePlus Real-Time PCR System (Applied Biosystems). For the quantification of microRNA expression, total RNA was reverse transcribed with pre-designed gene-specific Bulge-LoopTM RT primers (Ribobio) and quantitative real-time PCR was performed using Bulge-LoopTM miRNA qPCR Primer Sets (Ribobio) following the vendor’s manual. The primers for qPCR analysis of genes involved in this study can be found in Table S1.

### Bisulfite sequencing

The promoter methylation of *Oct4* and *Nanog* was analyzed for J1 mESCs, OSKME-4, OSKME-5 iPSCs, and MEFs. Briefly, genomic DNA (1 μg) is subjected to sodium bisulphite conversion using EpiTect Bisulfite Kit (Qiagen). Purified genomic fragments were amplified using AceTaq DNA Polymerase (Vazyme) through nested or semi-nested PCR reactions. The PCR products were then cloned into pGEM-T Easy vector (Promega), after which individual transformants were screened by colony PCR and positive colonies were sequenced. The sequencing results were analyzed with BiQ Analyzer . PCR primers used for bisulfite sequencing can be found in Table S1.

### Western blotting

Cells were washed cold PBS and lysed on ice for 15 min with RIPA buffer (Vazyme) supplemented with protease inhibitor cocktail (Roche), after which the cell lysate was collected and centrifuged to eliminate the cell debris. The supernatant mixed with loading buffer was boiled at 100 °C for 5 min and subjected to SDS-PAGE separation. The protein was then transferred to PVDF membrane (Invitrogen) and blocked with 5% nonfat dry milk (BD Biosciences) for 1 h at room temperature. The blot membrane was incubated with primary antibody overnight at 4 °C, washed three times with TBST buffer and incubated with HRP-conjugated secondary antibody for 1 h at room temperature. Signals were detected using High-sig ECL Western Blotting Substrate (Tanon). The antibodies used for immunoblotting were anti-TET2 (1:1500, 21207-1-AP, Proteintech) and anti-GAPDH (1:5000, KM9002, SUNGENE BIOTECH).

### Dot blot

Purified genomic DNA was denatured in 100 mM NaOH, 10 mM EDTA for 10 min at 95 °C and neutralized with 1 M NH_4_OAc on ice for 5 min, after which they were diluted to a final concentration of 25 ng μl^−^^1^ and then serially diluted. Equal volume of samples was spotted onto the pre-balanced nitrocellulose membrane. The membrane was incubated at 80 °C for 5 min and subjected to UV cross-link at 120,000 μJ cm^−^^2^. Then the membrane was blocked with 5% nonfat dry milk for 1 h at room temperature, incubated with anti-5hmC antibody (1:6000, 39791, Active Motif) overnight at 4 °C, and washed three times with TBST buffer. The membrane was next incubated with HRP-conjugated secondary antibody for 1 h at room temperature, and the signal was detected with ECL reagents. The same membrane was finally stained with 0.02% methylene blue for 10 min in 0.3 M sodium acetate (pH 5.2) to ensure equal spotting of the total genomic DNA on the membrane.

### EB differentiation

For mESCs or iPSCs cultured on feeder layers, colonies were trypsinized into single cells and transferred onto gelatin coated culture dish in standard mESCs medium, the cells were incubated at 37 °C for 45 min to eliminate the feeder cells, after which the floating cells were collected by centrifugation. Then 1.2 × 10^6^ cells were suspended with differentiation medium (standard mESCs medium without LIF) and transferred to 60 mm petri dish for further differentiation. The differentiation medium for EB culture was changed every 2 days. EB samples were collected on the indicated time point and lysed for characterization. For immunofluorescence staining, EBs were seeded into a gelatin coated 48-well plate by day 7 and cultured for another 6 days before fixation.

### Teratoma formation

Two lines of OSKME-iPSCs (OSKME-4, OSKME-5) and J1 mESCs were subjected to teratoma formation assay. To generate teratoma, cells were trypsinized and resuspended with serum-free culture medium. About 5 × 10^6^ cells in 100 μl media were injected subcutaneously at each site near dorsal flank of anesthetized nude mice (CD-1). Three replicates were performed for each cell line. Tumors were harvested 4 weeks later and fixed in 4% formaldehyde for 24 h at room temperature before paraffin embedding. For histological analysis, the specimens were stained with hematoxylin and eosin (HE). The use of mice in this study was approved by Animal Care and Use Committee, Northwest A&F University.

### Flow cytometry analysis

The cells were washed once with PBS and detached with Accutase (Gibco). After centrifugation, cells were washed twice with PBS and resuspended in staining buffer (2% FBS in PBS) for cell counting. About 1 × 10^6^ cells were then transferred into 1.5 ml Eppendorf tubes, centrifuged and incubated in 100 μl staining buffer supplemented with PE anti-mouse CDH1 antibody (1 μg, 147303, BioLegend) for 30 min on ice. The cells were washed twice and resuspended in 500 μl staining buffer and analyzed with FACSCalibur (BD Biosciences). The results were analyzed with FlowJo software (Tree Star).

### Cell proliferation assay

To investigate the effect of *Etv5* on cell proliferation in the reprogramming process, viable cells were counted directly. Briefly, two days after the final round of infection, cells were trypsinized and reseeded into wells of 12-well plates at a density of 5 × 10^3^ cells per well, after which the cells were counted daily from day 1 to day 5.

To investigate the effect of *Etv5* on self-renewal of mESCs, cell proliferation rate was assayed using CCK-8 Cell Counting Kit (Vazyme) according to the manufacturer’s instruction. Briefly, a total of 1.5 × 10^3^ cells were seeded into each well of a 48-well plate, and the medium was changed every 24 h. The CCK-8 solution (20 μl) was added to wells upon measurement, followed by 1 h incubation at 37 °C. The absorbance of the medium at 450 nm was then measured using Epoch™ Microplate Spectrophotometer (BioTek).

### Gene knockdown by shRNAs

For RNAi lentivirus packaging, the lentiviral vector pSicoR-puro (2 μg), psPAX2 (1.5 μg) and pMD2.G (1 μg) were co-transfected into HEK293T cells in 60 mm culture dishes using TurboFect Transfection Reagent (Thermo Scientific). The medium was changed 6 h later and the viral supernatant was collected at 48 h and filtered through 0.45 μm PVDF membrane. The lentiviruses were used to infect J1 mESCs which were seeded onto gelatin coated culture dishes 24 h earlier. Two rounds of infection with 12 h interval were performed. The KSR based mESCs medium supplemented with 1.5 μg ml^−^^1^ puromycin was used to screen the puro-resistant colonies.

### RNA-seq

Total RNA was isolated from shCtrl mESCs and *Etv5*-KD mESCs using RNAiso Plus (TaKaRa). Two independent batches of samples were collected for RNA-seq. For strand-specific library construction and sequencing, rRNAs were removed to retain mRNAs and ncRNAs, which were then fragmented and reverse transcribed into cDNA with random primers. Second-strand cDNA were synthesized using dUTP and purified with QiaQuick PCR extraction kit (Qiagen). Then the second-strand cDNA were end repaired, poly (A) added, and ligated to Illumina sequencing adapters. UNG (Uracil-N-Glycosylase) was next used to digest the second-strand cDNA. The digested products were size-selected by agarose gel electrophoresis and amplified by PCR. The sequencing was carried on Illumina HiSeq 4000. For bioinformatics analysis, raw reads containing adapter or of low quality (Q-value ≤ 20) were removed, then mapped to the mouse reference genome (GRCm38) by HISAT2 (version 2.1.0). The resulting files were sorted using SAMtools and subjected to HTSeq (version 0.9.0) to obtain the counts of each gene. For differential expression analysis, genes abundances were quantified by R package edgeR. FDR < 0.05 and log_2_ (fold change) > 1 were used as the threshold to define gene expression differences as significant. GO and KEGG analysis of the differentially expressed genes were performed using the R package clusterProfiler^28^. RNA-seq data of this study can be accessed with accession number SRP111429 in NCBI’s Sequence Read Archive. RNA-seq data of *Tet2*-KD mESCs were downloaded from GEO (GSE84458), which were processed by the same analysis procedure as *Etv5*-KD data did.

### Statistical analyses

The statistics methods used in this study include unpaired two-tailed Student’s *t* test, one-way ANOVA with Dunnett’s multiple comparisons test, and two-way ANOVA with Dunnett’s multiple comparisons test, and two-way ANOVA with Sidak’s multiple comparisons test. The application of each statistics method was specified in figure legends. GraphPad Prism 7.0 was used for the statistical analysis, and data are presented as means ± SD.

## Electronic supplementary material

Fig.S1

Fig.S2

Fig.S3

Fig.S4

Fig.S5

Fig.S6

Supplementary Table S1

Supplementary Figure Legends
