# Supplementary figures and images for: The oncogene Etv5 promotes MET in somatic reprogramming and orchestrates epiblast/primitive endoderm specification during mESCs differentiation
Source: Cell Death Dis. 2018 Feb 14;9(2):224. doi: 10.1038/s41419-018-0335-1 (PMC5833841; doi:10.1038/s41419-018-0335-1)

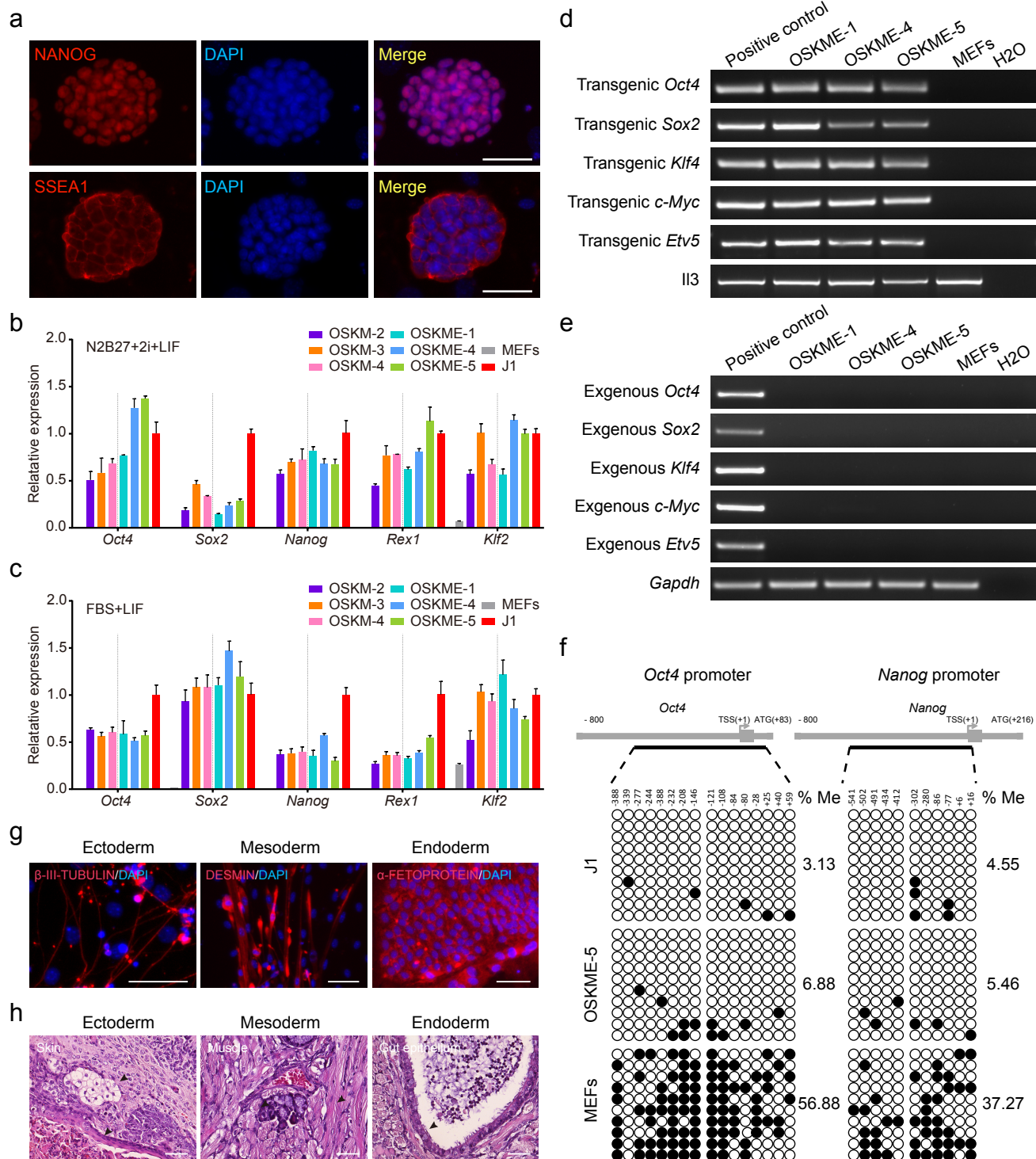

Supplemental Figure 1

Supplement: Supplementary file 1 — Fig.S1 [file 41419_2018_335_MOESM1_ESM.pdf]

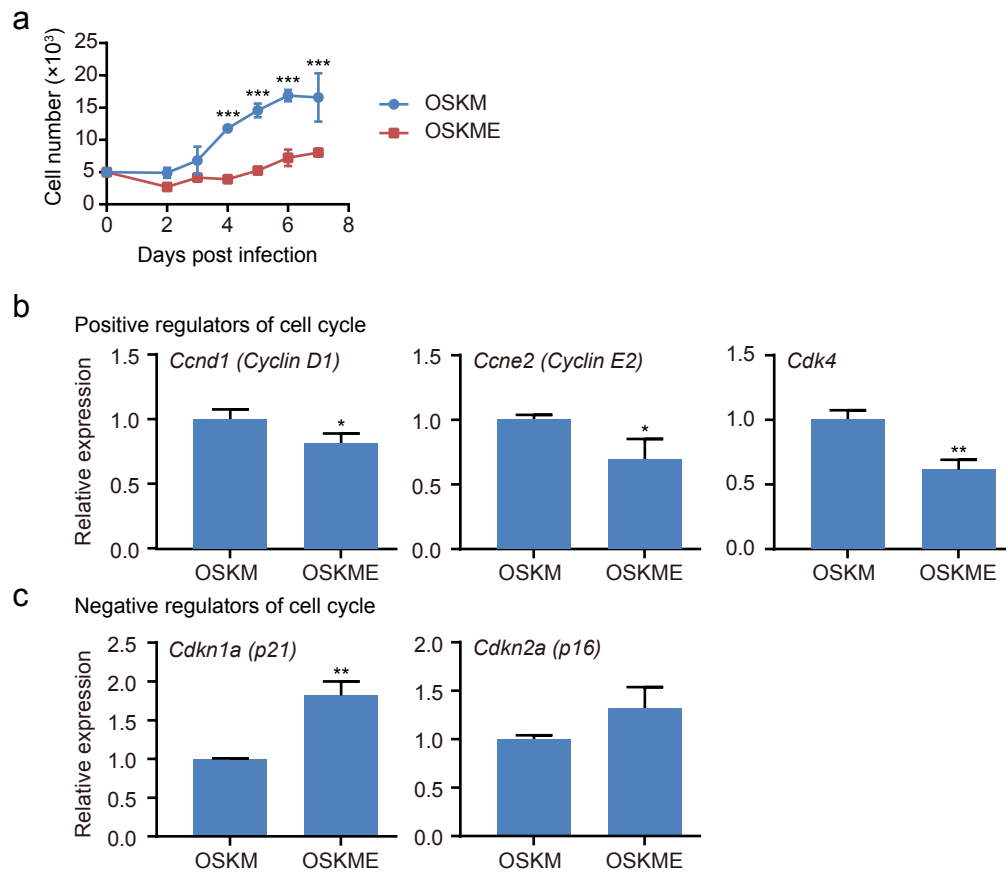

Supplemental Figure 2

Supplement: Supplementary file 2 — Fig.S2 [file 41419_2018_335_MOESM2_ESM.pdf]

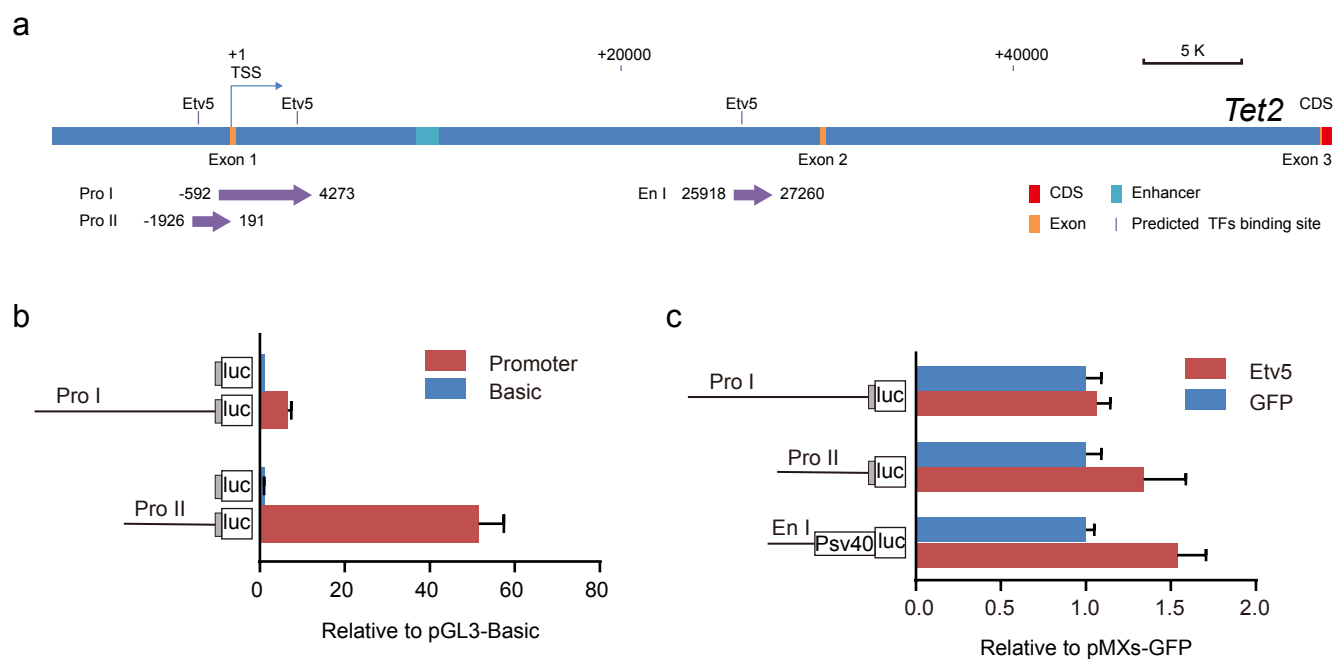

Supplemental Figure 3

Supplement: Supplementary file 3 — Fig.S3 [file 41419_2018_335_MOESM3_ESM.pdf]

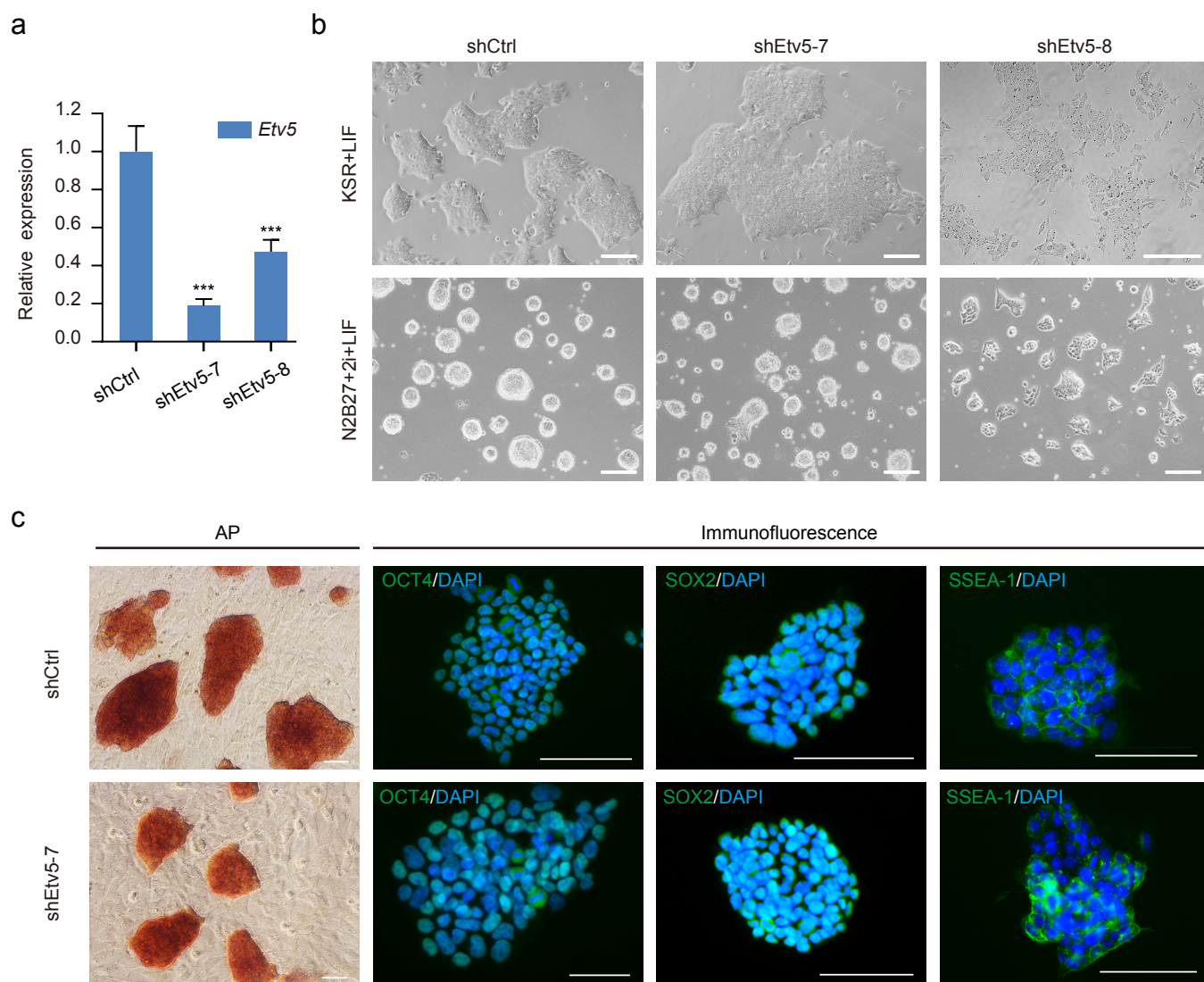

Supplemental Figure 4

Supplement: Supplementary file 4 — Fig.S4 [file 41419_2018_335_MOESM4_ESM.pdf]

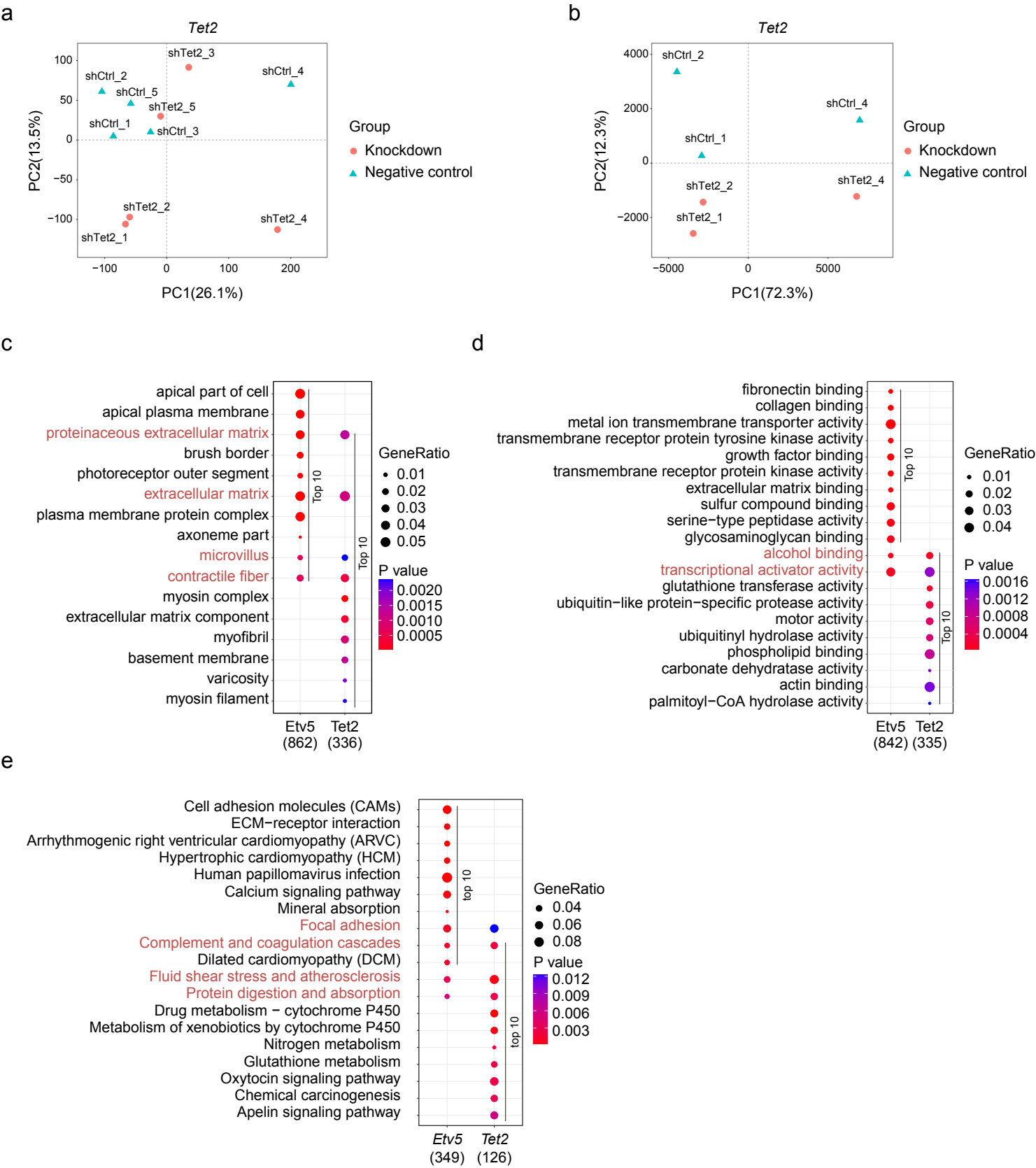

Supplemental Figure 5

Supplement: Supplementary file 5 — Fig.S5 [file 41419_2018_335_MOESM5_ESM.pdf]

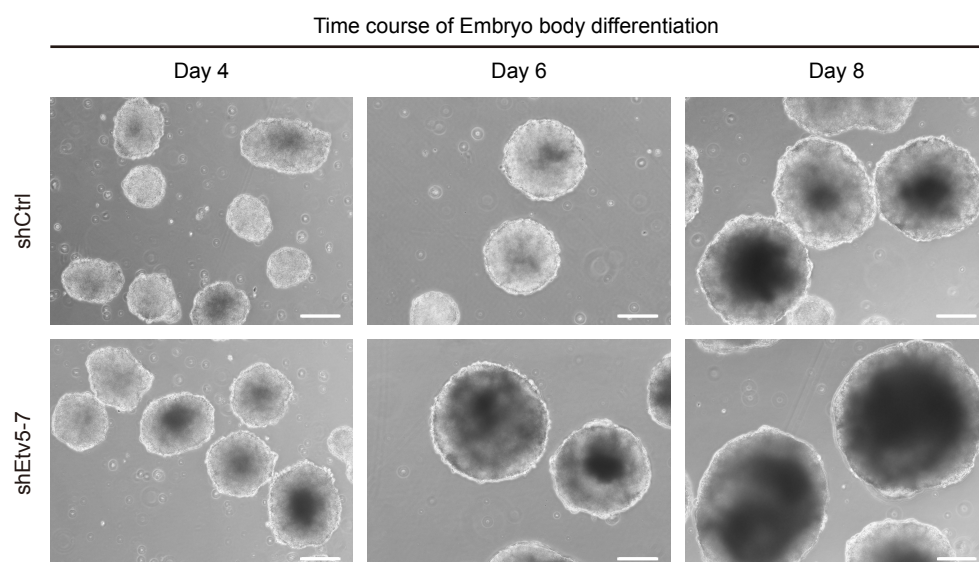

Supplemental Figure 6

Supplement: Supplementary file 6 — Fig.S6 [file 41419_2018_335_MOESM6_ESM.pdf]
