# Supplementary material for: The oncogene Etv5 promotes MET in somatic reprogramming and orchestrates epiblast/primitive endoderm specification during mESCs differentiation
Source: Cell Death Dis. 2018 Feb 14;9(2):224. doi: 10.1038/s41419-018-0335-1 (PMC5833841; doi:10.1038/s41419-018-0335-1)
Supplement: Supplementary file 7 — Supplementary Table S1 [file 41419_2018_335_MOESM7_ESM.pdf]

Supplementary Table S1. Primers used in this study

**Usage**	**Primer names**	**Sequences (5’---3’)**
Transcription Factors CDS cloning	mc-*Bcl6b*-P-1453-F	CCCaagcttGCCACCATGGGTTCCACAGCGG
	mc-*Bcl6b*-P-1453-R	CCGCGGctcgagACTATGGTCCCCCGAGGATG
	mc-*Etv5*-P-1559-F	CGggatccGCCACCATGGATGGGTTTTGTGATCAG
	mc-*Etv5*-P-1559-R	CCAGGctcgagATTAGTAAGCGAAGCCTTCGG
	mc-*Foxo1*-P-1986-F	TTggatccGCCACCATGGCCGAAGCGCCCCA
	mc-*Foxo1*-P-1986-R	GCTCActcgagATTAGCCTGACACCCAGCTGT
	mc-*Id4*-P-511-F	CCggatccGCCACCATGAAGGCGGTGAGCCC
	mc-*Id4*-P-511-R	TGCGctcgagATCAGCGGCAGAGAATGCTGT
	mc-*Lhx1*-P-1245-F	CCggatccGCCACCATGGTGCACTGTGCGGGCT
	mc-*Lhx1*-P-1245-R	CCGctcgagACTACCACACGGCTGCCTCGTTCA
	mc-*Plzf*-P-2048-F	CGggatccGCCACCATGGATCTGACAAAGATGG
	mc-*Plzf*-P-2048-R	GGCCGctcgagATCACACATAACACAGGTAG
	mc-*Taf4b*-P-2593-F	CGggatccGCCACCATGCCGGCGGGCCTCA
	mc-*Taf4b*-P-2593-R	GGCGctcgagATCACTTAAGAAGGGCAAGGTATA
Promoters & Enhancer cloning	mc-*Tet2*-ProI-NheI-4865-F	GCTAGCCCAGGCCCCTCTAGCTCTC
	mc-*Tet2*-ProI-XhoI-4865-R	CTCGAGTCCCCTGCCAGACCCTACACA
	mc-*Tet2*-ProII-XhoI-2132-F	CCCTCGAGACAGAGTCTAAGCCAACTGGTC
	mc-*Tet2*-ProII-HindIII-2132-R	CTCAAGCTTCTGGTTCTGGTCCCTCAGC
	mc-*Tet2*-EnI-MluI-1343-F	ATTACGCGTTGCTGGCTTTCAATGTTTAGACAC
	mc-*Tet2*-EnI-SalI-1343-R	ATTGTCGACGCATTTCATCCACCAAGCCATG
Genotyping and transgene silencing	pMXs-TgUS	GTGGTGGTACGGGAAATCAC
	pMXs-*Oct3/4*-TgDS	TAGCCAGGTTCGAGAATCCA
	pMXs-*Klf4*-TgDS	GGGAAGTCGCTTCATGTGAG
	pMXs-*Sox2*-TgDS	GGTTCTCCTGGGCCATCTTA
	pMXs-*c-Myc*-TgDS	AGCAGCTCGAATTTCTTCCA
	pMXs-*Etv5*-TgUS	TCCTCTAGACTGCCGGAT
	pMXs-*Etv5*-TgDS	AACTCTTCAGAATCGTGAGCC
RT-qPCR	mc-*Etv5*-Q-162-F	TCAGTCTGATAACTTGGTGCTTC
	mc-*Etv5*-Q-162-R	GGCTTCCTATCGTAGGCACAA
	mc-*Cdh1*-140-F	CAGCCTTCTTTTCGGAAGACT
	mc-*Cdh1*-140-R	GGTAGACAGCTCCCTATGACTG
	mc-*Zeb1*-Q-91-F	ACCGCCGTCATTTATCCTGAG
	mc-*Zeb1*-Q-91-R	CATCTGGTGTTCCGTTTTCATCA
	mc-*Zeb2*-Q-109-F	ATTGCACATCAGACTTTGAGGAA
	mc-*Zeb2*-Q-109-R	ATAATGGCCGTGTCGCTTCG
	mc-*Snai1*-133-F	CACACGCTGCCTTGTGTCT
	mc-*Snai1*-133-R	GGTCAGCAAAAGCACGGTT
	mc-*Tet1*-Q-112-F	ACACAGTGGTGCTAATGCAG
	mc-*Tet1*-Q-112-R	AGCATGAACGGGAGAATCGG
	mc-*Tet2*-Q-104-F	AGAGAAGACAATCGAGAAGTCGG
	mc-*Tet2*-Q-104-R	CCTTCCGTACTCCCAAACTCAT
	mc-*Tet3*-Q-188-F	CATGCCCTTGAGCTCCAACGA
	mc-*Tet3*-Q-188-R	AGTTGTGTTCACTGTCCGACCA
	mc-*Fgf5*-Q-191-F	GCTGTGTCTCAGGGGATTGT
	mc-*Fgf5*-Q-191-R	CACTCTCGGCCTGTCTTTTC
	mc-*T*-Q-148-F	CTCGGATTCACATCGTGAGAG
	mc-*T*-Q-148-R	AAGGCTTTAGCAAATGGGTTGTA
	mc-*Gata4*-Q-139-F	CCCTACCCAGCCTACATGG
	mc-*Gata4*-Q-139-R	ACATATCGAGATTGGGGTGTCT
	mc-*Gata6*-Q-164-F	GCGCCTCCTCTCTCCTTTTT
	mc-*Gata6*-Q-164-R	GCGCTACTCCAACCTGACTT
	mc-*Flk1*-Q-162-F	GGCGGTGGTGACAGTATCTT
	mc-*Flk1*-Q-162-R	GTCACTGACAGAGGCGATGA
	mc-*Pax6*-Q-194-F	TACCAGTGTCTACCAGCCAAT
	mc-*Pax6*-Q-194-F	TGCACGAGTATGAGGAGGTCT
	mc-*Oct3/4*(endo)-Q-212-F	TCCCTAGGTGAGCCGTCT
	mc-*Oct3/4*(endo)-Q-212-R	TACCTCTGAGCCTGGTCCGAT

To be continued

Supplementary Table S1. Primers used in this study(continued)

Usage	Primer names	Sequences (5'---3')
RT-qPCR	mc-Sox2(endo)-Q-297-F	TAGAGCTAGACTCCGGGCGATGA
	mc-*Sox2*(endo)-Q-297-R	TTGCCTTAAACAAGACCACGAAA
	mc-*Nanog*-Q-100-F	TCTTCCTGGTCCCCACAGTTT
	mc-*Nanog*-Q-100-R	GCAAGAATAGTTCTCGGGATGAA
	mc-*Rex1*-Q-112-F	CCCTCGACAGACTGACCCTAA
	mc-*Rex1*-Q-112-R	TCGGGGCTAATCTCACTTTCAT
	mc-*Klf2*-Q-133-F	TCGAGGCTAGATGCCTTGTGA
	mc-*Klf2*-Q-133-R	AAACGAAGCAGGCGGCAGA
	mc-*Ccnd1*-Q-183-F	GCGTACCCTGACACCAATCTC
	mc-*Ccnd1*-Q-183-R	CTCCTCTTCGCACTTCTGCTC
	mc-*Ccne2*-Q-198-F	ATGTCAAGACGCAGCCGTTTA
	mc-*Ccne2*-Q-198-R	GCTGATTCCTCCAGACAGTACA
	mc-*Cdk4*-Q-129-F	ATGGCTGCCACTCGATATGAA
	mc-*Cdk4*-Q-129-R	TCCTCCATTAGGAACTCTCACAC
	mc-*p16*-Q-200-F	AGAGCGGGGACATCAAGAC
	mc-*p16*-Q-200-R	AGAAAAAGGCGGGCTGAG
	mc-*p21*-Q -61-F	CCTGGTGATGTCCGACCTG
	mc-*p21*-Q -61-R	CGGGACCGAAGAGACAACG
	mc-*Gapdh*-Q-123-F	AGGTCGGTGTGAACGGATTTG
	mc-*Gapdh*-Q-123-R	TGTAGACCATGTAGTTGAGGTCA
Bisulfate sequencing	*Oct4*-outer-F	GAGGATTGGAGGTGTAATGGTTGTT
	Oct4-outer-R	CTACTAACCCATCACCCCCACCTA
	Oct4-inner-F	CAAGCTTTGGGTTGAAATATTGGGTTTATTT
	Oct4-inner-R	CGGATCCCTAAAACCAAATATCCAACCATA
	Nanog-outer-F	AAGTATGGATTAATTTATTAAGGTAGTT
	Nanog-outer-R	AAAAAACCCACACTCATATCAATATA
	Nanog-inner-F	AAGTATGGATTAATTTATTAAGGTAGTT
	Nanog-inner-R	CAACCAAATCAACCTATCTAAAAA

F, forward; R, reverse
