# Supplementary material for: The oncogene Etv5 promotes MET in somatic reprogramming and orchestrates epiblast/primitive endoderm specification during mESCs differentiation
Source: Cell Death Dis. 2018 Feb 14;9(2):224. doi: 10.1038/s41419-018-0335-1 (PMC5833841; doi:10.1038/s41419-018-0335-1)
Supplement: Supplementary file 8 — Supplementary Figure Legends [file 41419_2018_335_MOESM8_ESM.docx]

**Supplementary Figure Legends**

**Fig. S1. Pluripotency Characterization of OSKME-iPSCs**

**a** Immunostaining of NANOG (red) and SSEA-1 (red) for OSKME-iPSCs. Nuclei were counterstained with DAPI (blue). Scale bars, 50μm.

**b***,* **c** RT-qPCR analysis of *Oct4*, *Sox2*, *Nanog*, *Rex1* and *Klf2* for cell lines of OSKME-iPSCs (OSKME-1, -4 and -5). The cell lines of OSKM-iPSCs (OSKM-2, -3 and -4) are compared in parallel. MEFs is used as negative control. J1 mESCs is used as positive control. These cells cultured in 2i medium (N2B27+2i+LIF) and standard mESCs medium are both investigated. Data are shown as mean ± SD (n=3).

**d** Genomic integration of exogenous genes in cell lines of OSKME-iPSCs (OSKME-1, -4 and -5). MEFs infected with OSKME at 5 dpi are used as positive control. MEFs (passage 2) and H_2_O are used as the negative control. *Il3* is used as internal control of genomic RT-PCR.

**e** Analysis of exogenous genes silencing in cell lines of OSKME-iPSCs (OSKME-1, -4 and -5). Control settings are the same as **d**; *Gapdh* was used as the internal control of semi-RT-PCR.

**f** Bisulfite sequencing of *Oct4* and *Nanog* promoter for cell line of OSKME-iPSCs (OSKME-5). MEFs is used as negative control. J1 mESCs is used as positive control. Open and filled circles indicate unmethylated and methylated CpG dinucleotides, respectively. The percentage of methylated sites (% Me) is also presented.

**g** Immunostaining of markers representing three germ layers from OSKME-iPSCs-derived embryoid bodies (EB). The markers (red) of ectoderm (β-III-TUBULIN), mesoderm (DESMIN) and endoderm (α-FETOPROTEIN) are shown. Nuclei were counterstained with DAPI (blue). Scale bars, 50μm.

**h** Hematoxylin-eosin (HE) staining of teratomas generated from OSKME-iPSCs. Representative structures of ectoderm (squamous epithelium and sebaceous gland), mesoderm (skeletal muscle) and endoderm (glandular epithelial) are marked with arrowhead. Scale bars, 50 μm.

**Fig. S2. The Roles of Cellular Proliferation in OSKME-mediated Reprogramming**

**a** Growth curves of MEFs infected with OSKME and OSKM. Data are shown as mean ± SD (n=3). Two-way ANOVA with Sidak's multiple comparisons test was used. ***P < 0.001.

**b** RT-qPCR analysis of cell cycle positive regulators (*Ccnd1*, *Ccne2* and *Cdk4*) in MEFs infected with OSKM and OSKME at 5 dpi.

**c** RT-qPCR analysis of cell cycle negative regulators (*Cdkn1a*, *Cdkn2a*) in MEFs infected with OSKM and OSKME at 5 dpi. Data are shown as mean ± SD (n=3). Student’s t test was used in **b** and **c**.* P<0.05, **P<0.01.

**Figure S3. Luciferase assay of *Etv5* on *Tet2* Promoter and Enhancer**

**a** The diagram of predicted *Etv5* binding sites upstream of *Tet2*. Three fragments (Pro I, Pro II, and En I) were cloned for testing luciferase activity. TSS, Transcription start site. CDS, Coding sequence.

**b** Promoter activity analysis showing the normalized luciferase expression of Pro I and Pro II in NIH3T3 cells. pGL3-Basic was set as 1. Data are shown as mean ± SD (n=3).

**c** Normalized luciferase expression of co-transfection of *Etv5* with Pro I , Pro II and En I in NIH3T3 cells. Co-transfection of GFP with Pro I , Pro II and En I was set as 1. Data are shown as mean ± SD (n=3).

**Fig. S4. Phenotype of *Etv5*-KD mESCs**

**a** RT-qPCR verification of *Etv5* expression in mESCs stably infected with shCtrl and *Etv5* shRNAs (shEtv5-7 and shEtv5-8). Data are shown as mean ± SD (n=3), One-way ANOVA with Dunnett’s multiple comparisons test, ***P < 0.001.

**b** Morphology of mESCs infected with shCtrl and *Etv5* shRNAs (shEtv5-7 and shEtv5-8). These morphology of cells cultured in modified mESCs medium (KSR+LIF) and 2i medium (N2B27+2i+LIF) are shown.

**c** AP staining (red) and immunostaining (green) of OCT4, SOX2 and SSEA-1 for mESCs infected with shCtrl and shEtv5-7. Nuclei were counterstained using DAPI (blue). Scale bars, 50 μm.

**Fig. S5. Principal Component Analysis (PCA) and Gene Ontology (GO) Analysis**

**a** PCA of the global gene expression pattern of *Tet2*-KD mESCs and their corresponding control mESCs. Five biological replicates were shown. The percentage of variance explained by each principal component is indicated in parentheses.

**b** PCA of the global gene expression pattern of filtered replicates from *Tet2*-KD mESCs and their corresponding control mESCs.

**c** GO analysis of differentially expressed genes in *Etv5*-KD mESCs (862 annotated genes, left column) and *Tet2*-KD mESCs (336 annotated genes, right column). The top 10 GO terms of cellular components were plotted for *Etv5*-KD mESCs and *Tet2*-KD mESCs from each end of the graph according to their P values ranking. The GO terms from the other group overlapped with the top 10 GO terms are are also plotted and marked in red. The color of circles indicates the P value. The size of circles indicates the Gene Ratio. The same way of presentation is applied in **d** and **e**.

**d** GO analysis (molecular functions) of differentially expressed genes in *Etv5*-KD mESCs (842 annotated genes, left column) and *Tet2*-KD mESCs (335 annotated genes, right column).

**e** KEGG analysis of differentially expressed genes in *Etv5*-KD mESCs (349 annotated genes, left column) and *Tet2*-KD mESCs (126 annotated genes, right column).

**Fig. S6. EB Differentiation of mESCs Infected with shCtrl and shEtv5-7**

The time course (Day 4-8) images of EB generated from mESCs infected with shCtrl and shEtv5-7. Scale bar, 200 μm.
